# Complete mitochondrial genome of six *Cheilinus undulatus* (Napoleon Wrasse): an endangered marine fish species from Sabah, Malaysia

**DOI:** 10.1080/23802359.2018.1473725

**Published:** 2018-10-12

**Authors:** Pressly Matthew, B. Mabel Manjaji-Matsumoto, Kenneth Francis Rodrigues

**Affiliations:** a Endangered Marine Species Research Unit, Borneo Marine Research Institute, Universiti Malaysia Sabah, Kota Kinabalu, Malaysia;; b Biotechnology Research Institute, Universiti Malaysia Sabah, Kota Kinabalu, Malaysia

**Keywords:** Mitochondrial genome, *Cheilinus undulatus*, Malaysia, fish conservation

## Abstract

We report here the complete mitochondrial (mt) genomes of six individuals of *Cheilinus undulatus* (Napoleon Wrasse), an endangered marine fish species. The six mt DNA sequences had an average size of 17,000 kb and encoded 22 tRNA, two sRNA, 13 highly conserved protein coding genes and a control region. The polymorphic variation (control region) in these six individuals suggests their potential use as a specific marker for phylogeographic conservation. Moreover, the sequence polymorphism within the control region (D-loop) suggests that this locus can be applied for phylogenetic studies.

This is the first study of mitochondrial (mt) genome for endangered marine fish species *Cheilinus undulatus* from Sabah, Malaysia waters. *Cheilinus undulatus* is listed on the International Union for Conservation of Nature (IUCN) Red List of Threatened Species, and is protected by the Convention on International Trade in Endangered Species of Wild Fauna and Flora (CITES) Appendix 2. In the last few decades, the worldwide population of *C. undulatus* has declined. In Sabah State, targeted and overfishing activities to fulfil the local seafood demand (Scales et al. 2007) and international market (Sadovy et al. 2003) have contributed to this decline. The realization of the imminent loss of this fisheries resource has prompted the Malaysian government to regulate its international trade by listing the species on CITES Appendix 2 in 2014 (Fabinyi et al. 2014). Such listing effectively bans the international export of *C. undulatus*.

Here, as a part our efforts to establish specific fish phytogeography markers, we sequenced the full length mitochondrial DNA (mtDNA) of *Cheilinus undulatus*, collected from two geographic points: M3ss and M8ss (obtained from Sandakan (5.839°N, 118.117°E), Sabah, Malaysia) and M11kk, M12kk, M15kk, and M17kk (obtained from Kota Kinabalu (5.980°N, 116.073°E), Sabah, Malaysia). The assembly reads generated by MiSeq Illumina pair-end fastaq were trimmed, automated error correction and assembled using the SPAde *De Novo* Assembler (Bankevich et al. 2012). Mis-assemblies were corrected using Geneoius version 11.0.5 (Kearse et al. 2012). The mtDNA genome was annotated using MITOFish online annotation pipeline (Iwasaki et al. 2013). All six mtDNA were aligned using clustalW in MEGA 6 (Tamura et al. 2013) software with the two references of mtDNA of *C. undulatus* (accession number GU296101.1 and KM17184.1) which was previously published (Qi et al. 2013; Han et al. 2016).

The MiSeq Illumina platform yielded an average 85,000 paired reads for all six individuals. The length of the mtDNA of M3ss ( MH675879 ), M8ss (MH675880), M11kk (MH688049 ), and M15kk (MH688051) were 17,185 bp whereas, M12kk ( MH688050) and M17kk (MH688052) were found to be 17,184 bp. The G + C content was 47.5% and 47.6%, respectively. A total of 38 genes were identified which consisted of two rRNAs (*12sRNA* and *16sRNA*), 22 tRNA, 13 highly conserved respiratory genes, and a control region (D-loop). The gene arrangement was found to be similar with the gene arrangement for animal mt (Lee et al. 1995; Boore 1999). Notably, sequence polymorphisms were observed in all six individuals when aligned with the reference genome (accession number GU296101.1 and KM17184.1) especially in the control region which spans a region of 903 basepairs. This region can be applied to detect polymorphisms within individuals for the subsequent development of molecular markers. The evolutionary phylogenetic tree history of *Cheilinus undulatus* was inferred using the neighbour-joining method (Saitou and Nei 1987) using MEGA6 software (Tamura et al. 2013) (Figure 1).

**Figure 1.**
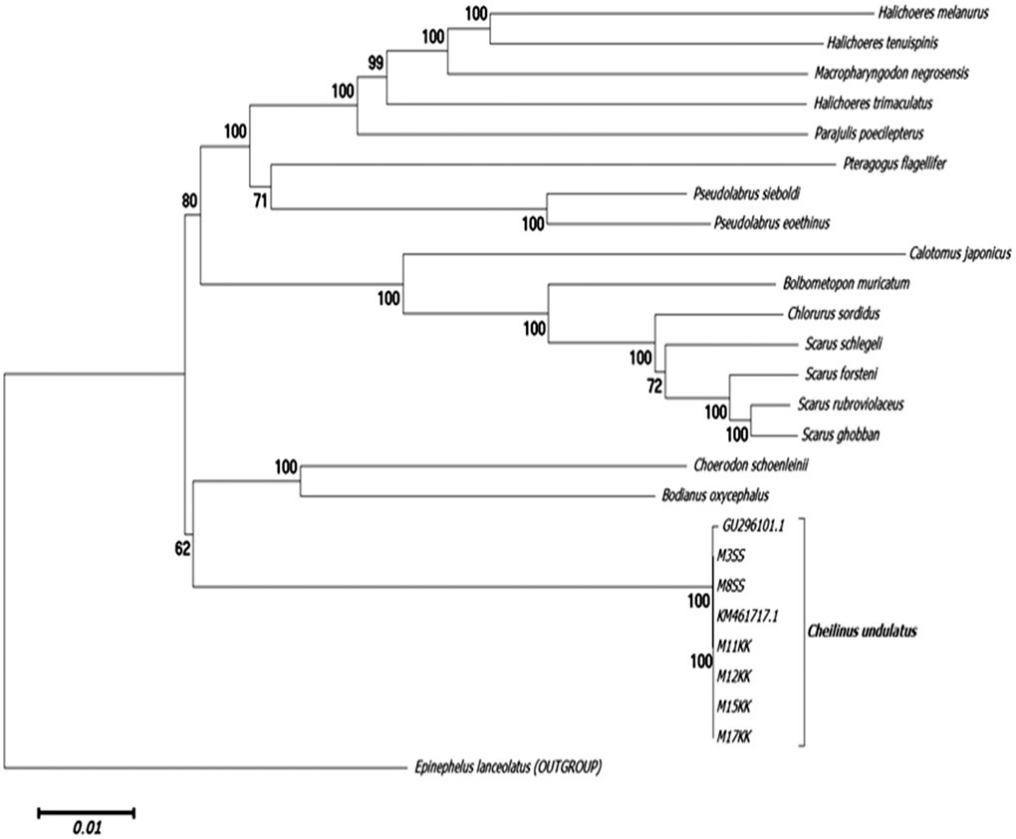
Molecular phylogeny of *Cheilinus undulatus* and 17 other Labridae species based on complete mitochondrial genome using neighbour-joining method (1000 bootstrap). The complete mitochondrial genome is downloaded from NCBI database and the phylogeny tree is constructed by MEGA6 software. The accession number for each gene used in the tree construction is listed as follows: *Halichoeres melanurus* (AP006018.1), *H. tenuispinis* (EU082205.1), *H. trimaculatus* (EU087704.1), *Macropharyngodon negrosensis* (KP013102.1), *Parajulis poecilepterus* (EF192032.2), *Pteragogus flagellifer* (EF409976.2), *Pseudolabrus sieboldin* (AP006019.1), *P. eoethinus* (EU560728.1), *Calotomus japonicus* (AP017568.1), *Bolbometopon muricatum* (KY235362.1), *Chlorurus sordidus* (AP006567.1), *Scarus schlegeli* (FJ595020.1), *S. forsteni* (FJ619271.1), *S. rubroviolaceus* (FJ227899.1), *S. ghobban* (FJ449707.1), *Choerodon schoenleinii* (KM487697.1), *Bodianus oxycephalus* (KT591189.1), *Cheilinus undulatus* (GU296101.1, KM461717.1). *Epinephelus lanceolatus* (FJ472837.1) was used as the outgroup.

## References

[CIT0001] Bankevich A, Nurk S, Antipov D, Gurevich AA, Dvorkin M, Kulikov AS, Pevzner PA. 2012 SPAdes: a new genome assembly algorithm and its applications to single-cell sequencing. J Comput Biol. 19:455–477. 22506599 10.1089/cmb.2012.0021 PMC3342519

[CIT0002] Boore JL. 1999 Animal mitochondrial genomes. Nucleic Acids Res. 27:1767–1780. 10101183 10.1093/nar/27.8.1767 PMC148383

[CIT0003] Fabinyi M, Pido M, Ponce de Leon EM, De las Alas MA, Buenconsejo J, Uyami-Bitara A, Caceres J. 2014 Fisheries trade and social development in the Philippine‐Malaysia maritime border zone. Dev Policy Rev. 32:715–732.

[CIT0004] Han Y, Chen G, Luo J, Wen X, Li W, Wang J. 2016 The complete mitochondrial genome of *Cheilinus undulates* based on high-throughput sequencing technique. Mitochondrial DNA A DNA Mapp Seq Anal. 27:1897–1899. 25329275 10.3109/19401736.2014.971276

[CIT0005] Iwasaki W, Fukunaga T, Isagozawa R, Yamada K, Maeda Y, Satoh TP, Nishida M. 2013 MitoFish and MitoAnnotator: a mitochondrial genome database of fish with an accurate and automatic annotation pipeline. Mol Biol Evol. 30:2531–2540. 23955518 10.1093/molbev/mst141 PMC3808866

[CIT0006] Kearse M, Moir R, Wilson A, Stones-Havas S, Cheung M, Sturrock S, Thierer T. 2012 Geneious basic: an integrated and extendable desktop software platform for the organization and analysis of sequence data. Bioinformatics. 28:1647–1649. 22543367 10.1093/bioinformatics/bts199 PMC3371832

[CIT0007] Lee WJ, Conroy J, Howell WH, Kocher TD. 1995 Structure and evolution of teleost mitochondrial control regions. J Mol Evol. 41:54–66. 7608989 10.1007/BF00174041

[CIT0008] Qi XZ, Yin SW, Luo J, Huo R. 2013 Complete mitochondrial genome sequence of the humphead wrasse, *Cheilinus undulatus*. Genet Mol Res. 12:1095–1105. 23661435 10.4238/2013.April.10.5

[CIT0009] Sadovy Y, Donaldson T, Graham T, McGilvray F, Muldoon G, Phillips M, Rimmer M, Smith A, Yeeting B 2003. While stocks last: the live reef food fish trade. Manila, Philippines: Asian Development Bank p. 19–20. [accessed 2018 June 30]. https://think-asia.org/handle/11540/2432

[CIT0010] Saitou N, Nei M. 1987 The neighbor-joining method: a new method for reconstructing phylogenetic trees. Mol Biol Evol. 4:406–425. 3447015 10.1093/oxfordjournals.molbev.a040454

[CIT0011] Scales H, Balmford A, Manica A. 2007 Impacts of the live reef fish trade on populations of coral reef fish off Northern Borneo. Proc R Soc Lond B: Biol Sci. 274:989–994. 10.1098/rspb.2006.0280 PMC2141675 17251096

[CIT0012] Tamura K, Stecher G, Peterson Filipski A, Kumar S. 2013 MEGA6: molecular evolutionary genetics analysis version 6.0. Mol Biol Evol. 30:2725–2729. 24132122 10.1093/molbev/mst197 PMC3840312

